# Comprehensive nursing care for advanced digestive malignancy patients during chemotherapy based on probiotic intervention: a randomized controlled study on improvement of gastrointestinal function and prevention of complications

**DOI:** 10.3389/fcimb.2025.1718665

**Published:** 2026-01-06

**Authors:** Feng Huang, Qi Zhuo, Lijuan Zhang, Zheng Gao, Chennuo He, Lanlan Zhang

**Affiliations:** Department of Day Treatment Room, Clinical Oncology School of Fujian Medical University, Fujian Cancer Hospital, National Health Commission Key Laboratory of Cancer Metabolism, Fuzhou, Fujian, China

**Keywords:** probiotics, dvanced digestive malignancy patients, chemotherapy, comprehensive nursing, gastrointestinal function, complications

## Abstract

**Objective:**

This study aims to investigate the effects of a comprehensive nursing protocol combined with a probiotic intervention on improving gastrointestinal function, reducing non-infectious complications, and enhancing the quality of life in patients with advanced digestive malignancies undergoing chemotherapy, thereby providing evidence-based support for chemotherapy nursing care.

**Methods:**

A total of 300 advanced digestive malignancy patients undergoing chemotherapy at Our Oncology Specialty Hospital from January 2021 to December 2024 were randomly divided into a control group (150 patients) and an observation group (150 patients) using a random number table. The control group received conventional chemotherapy nursing care, while the observation group received a combined probiotic intervention along with comprehensive nursing care. Baseline data, gastrointestinal function scores (bloating, diarrhea, constipation, nausea/vomiting), nutritional status indicators (serum albumin, prealbumin, BMI), and quality of life scores (EORTC QOLQ-C30) were recorded before and after 2, 4, and 8 weeks of intervention. Non-infectious complications and nursing satisfaction were also evaluated. The dynamic effects of the intervention were analyzed using Generalized Estimation Equations (GEE).

**Results:**

No significant differences were observed between the groups at baseline (P>0.05). However, after 2, 4, and 8 weeks of intervention, the observation group exhibited significantly improved gastrointestinal function, with lower scores across all dimensions compared to the control group (P<0.05). At week 8, the total gastrointestinal function score in the observation group (1.57 ± 0.58) was significantly lower than the control group (3.74 ± 1.05) (t=22.156, P = 0.000). The observation group also showed significantly higher nutritional indicators: serum albumin (41.53 ± 2.96 vs. 36.18 ± 3.42), prealbumin (276.41 ± 17.53 vs. 218.65 ± 20.37), and BMI (23.58 ± 2.86 vs. 22.37 ± 3.21) (P<0.01). Additionally, the observation group had higher quality of life scores and lower complication rates (P<0.05), with increased nursing satisfaction. GEE analysis confirmed that the observation group showed greater improvements in all measured indicators over time compared to the control group (P<0.01).

**Conclusion:**

The comprehensive nursing protocol with probiotic intervention significantly improves gastrointestinal function, enhances nutritional status, reduces non-infectious complications, and boosts the quality of life in chemotherapy patients with advanced digestive malignancy. This intervention demonstrates substantial clinical benefits and therapeutic value.

## 1 Introduction

In patients with Advanced digestive malignancies (including gastric, colorectal, and esophageal cancers), chemotherapy often causes severe gastrointestinal dysfunction. The core mechanisms involve direct toxicity of chemotherapeutic agents to intestinal epithelial cells, disruption of intestinal barrier function due to microbial imbalance, and gastrointestinal motility disorders caused by neuroendocrine dysfunction (Heindryckx and Sjöblom, 2024; Sorbye et al., 2013). Clinical data show that approximately 70% - 90% of patients experience symptoms such as abdominal distension, diarrhea, nausea, and vomiting during chemotherapy. Among these, 25% to 40% develop malnutrition (serum albumin < 35 g/L), while 15% to 20% require treatment discontinuation due to electrolyte imbalances or bowel obstruction (Watanabe et al., 2016). This vicious cycle of “symptom-nutrition-treatment” triggered by gastrointestinal dysfunction not only reduces patients’ quality of life but also significantly shortens their progression-free survival (Fijlstra et al., 2015). Chemotherapy for advanced digestive malignancies often leads to severe gastrointestinal dysfunction due to mucosal injury, microbial imbalance, and systemic inflammation, which collectively diminish patients’ nutritional status and quality of life. Despite advances in oncology nursing care, most conventional protocols focus on symptomatic relief rather than the underlying pathophysiological disruptions caused by gut microbiota imbalance (Łaniewski et al., 2020). Recent years have seen probiotics emerge as a research hotspot in adjuvant cancer therapy. In recent years, probiotic-based interventions have emerged as promising adjunctive strategies to modulate intestinal flora, enhance mucosal repair, and alleviate treatment-induced toxicity. However, existing research remains fragmented, as many studies examine isolated probiotic strains or short-term effects without integrating comprehensive, multidisciplinary nursing care approaches (Bender et al., 2023; Wu et al., 2018). To address this gap, the present study investigates the combined efficacy of probiotic intervention and comprehensive nursing management in patients with advanced digestive malignancy undergoing chemotherapy. The primary objective is to evaluate improvements in gastrointestinal function, nutritional status, and quality of life while reducing non-infectious complications through a quantitatively assessed, evidence-based clinical protocol. This study enrolled 300 patients with advanced digestive malignancies undergoing chemotherapy at our hospital from January 2021 to December 2024. Using a randomized controlled trial design, we compared the efficacy of conventional care with a “probiotic intervention + comprehensive care” protocol. Through monitoring gastrointestinal function scores, nutritional parameters (serum albumin, prealbumin, BMI), quality of life (EORTC QOLC-30), and non-infectious complications (including intestinal obstruction and electrolyte imbalances), we analyzed the dynamic effects of intervention methods on these indicators using generalized estimating equations. The findings aim to clarify the clinical value of this comprehensive approach. These results provide new strategies to break the vicious cycle of chemotherapy-related gastrointestinal dysfunction, offer evidence-based support for precision care in patients with advanced digestive malignancies, ultimately prolong effective treatment duration, and improve patient prognosis.

## 2 Data and methods

### 2.1 General information

This study enrolled 300 patients with advanced digestive malignancies undergoing chemotherapy at our Oncology Specialty Hospital between January 2021 and December 2024. The protocol was approved by the Medical Ethics Committee of our hospital, adhering strictly to the Helsinki Declaration and international guidelines for medical research ethics. All patients and their legal guardians were fully informed of the study details and provided written consent, with the right to withdraw at any time without affecting subsequent standard treatments. (1) Inclusion criteria: ① Confirmed by surgical histopathology or biopsy cytology as Advanced digestive malignancy patients (including gastric cancer, colorectal cancer, esophageal cancer) meeting International Union Against Cancer (UICC) stage IV diagnosis (Vuity et al., 2018); ② Planned to receive a chemotherapy regimen combining fluorouracil-based drugs with platinum-based agents, with a planned treatment duration ≥8 weeks; ③ Age 18–75 years, gender unrestricted; ④ Karnofsky Physical Status Score (KPS) ≥60 (Agarwal et al., 2017); ⑤ Estimated survival ≥3 months based on imaging assessment and clinical judgment; ⑥ No history of major gastrointestinal surgery (e.g., subtotal gastrectomy, small bowel resection>50%), or postoperative recovery ≥6 months with stable gastrointestinal function; ⑦ Patients were conscious with basic communication ability, and family members could assist in daily symptom documentation and follow-up; ⑧ No participation in other similar clinical trials within the past 3 months. (2) Exclusion Criteria: ① Severe hepatic or renal impairment: Child-Pugh Class C liver function, or serum creatinine>200μmol/L with endogenous creatinine clearance <30mL/min; ② Absolute contraindications to chemotherapy: Uncontrolled intestinal obstruction (imaging showing bowel dilation with air-fluid level), major gastrointestinal hemorrhage within 1 month (blood loss>500mL) or coagulation disorder (INR>1.5); ③ Recent use of probiotics, or systemic antibiotics (Course of treatment ≥7 days) that may interfere with gut microbiota assessment; ④ Known hypersensitivity to probiotic components or dairy products; ⑤ Concurrent psychiatric disorders (e.g., schizophrenia), cognitive impairment, or poor clinical compliance; ⑥ Presence of severe underlying diseases; ⑦ Existing severe malnutrition or cachexia at enrollment. Grouping was conducted using a randomized number table method: Statistical professionals generated a random sequence of 1–300 numbers, which were assigned sequentially according to patient enrollment order. Odd-numbered individuals were allocated to the observation group, while even-numbered individuals were assigned to the control group, with 150 cases in each group. The grouping process was concealed using a sealed envelope method until data collection was completed.

### 2.2 Research methods

Both patient groups received standardized chemotherapy regimens based on tumor type: Gastric cancer patients received the SOX regimen (Oxaliplatin 130 mg/m² intravenous infusion, day 1; Tegafur 40-60mg orally twice daily, days 1-14); Colorectal cancer patients received the FOLFOX regimen (Oxaliplatin 85 mg/m² intravenous infusion, day 1; Leucovorin Calcium 400 mg/m² intravenous infusion, day 1; Fluorouracil 400 mg/m² intravenous bolus, followed by continuous 46-hour infusion of 2400 mg/m²); Esophageal cancer patients received the TP regimen (Paclitaxel 175 mg/m² intravenous infusion, day 1; Carboplatin 75 mg/m² intravenous infusion, day 1). All regimens followed a 2-week cycle, with four complete cycles (8 weeks) of treatment. Standard supportive care included antiemetics (Palonosetron 0.25mg intravenous injection, day 1) and gastric protection (Pantoprazole 40mg intravenous infusion once daily).

#### 2.2.1 Control group

Conventional Chemotherapy Nursing Protocol: (1) Health Education: One day before chemotherapy, the assigned nurse conducts one-on-one education using a color-coded illustrated manual (including chemotherapy flowcharts and gastrointestinal reaction grading standards), focusing on explaining the mechanisms of nausea/vomiting caused by fluorouracil drugs and diarrhea induced by platinum-based medications, along with home management strategies. Daily 30-minute group lectures feature case videos to reinforce patient understanding, supplemented by the distribution of the “Home Care Manual for Chemotherapy” for reference. (2) Dietary Guidance: Based on China’s Dietary Guidelines, a universal dietary plan is formulated: Daily protein intake should be 1.2-1.5g/kg (premium protein ratio ≥60%, e.g., eggs, fish), 20-30g dietary fiber (e.g., leafy greens, whole grains), avoiding spicy, fried, and gas-producing foods (e.g., chili peppers, fried dough sticks, onions). Daily water intake should be controlled at 1500-2000mL (divided into multiple small sips, avoiding large amounts on an empty stomach or within 1 hour after meals), with 24-hour fluid intake/output recorded. (3) Symptom Management: A graded symptom management protocol is established: ① Nausea/vomiting: Grade I (occasional nausea): Oral vitamin B6 tablets (10mg, 3 times daily); Grade II (1–2 daily vomiting episodes): Metoclopramide injection (10mg, intramuscular injection, repeat every 4–6 hours if necessary); Grade III (≥3 daily vomiting episodes): Promptly report to the physician for adjustment of antiemetic therapy. ② Diarrhea: Grade I (loose stools 1–2 times daily) -Montmorillonite Powder (3g,3 times daily, taken on an empty stomach); Grade II (loose stools 3–4 times daily) -Oral rehydration solution (III) (1 bag dissolved in 500mL warm water, divided into multiple doses); Grade III (loose stools ≥5 times daily) -Suspend chemotherapy and administer intravenous fluid replacement. ③ Constipation: Grade I (defecation every 2–3 days) -Lactulose oral solution (15mL, twice daily); Grade II (defecation every 4–5 days) -glycerin enema (20mL, administered rectally); Grade III (no bowel movement for ≥6 days) -Consult the physician to determine whether a cleansing enema is required. (4) Psychological Support: A psychological counselor provides each patient with a 20-minute one-on-one counseling session. Cognitive behavioral therapy (e.g., correcting the negative belief that “vomiting means treatment failure”) and relaxation techniques (deep breathing, progressive muscle relaxation). Tumor patient support groups may be recommended when necessary.

#### 2.2.2 Observation group

Probiotic Intervention Combined with Comprehensive Nursing Protocol: (1) Probiotic Intervention ① Preparation Selection: Bifidobacterium Triple Live Capsules, containing ≥0.5×10^^7^ CFU Bifidobacterium, ≥0.5×10^^7^ CFU Lactobacillus acidophilus, ≥0.5×10^^7^ CFU Enterococcus faecalis, with total live bacteria count ≥1.5×10^^7^ CFU/g per capsule. ② Dosage and Administration: Take 4 capsules at a time daily, dissolved in warm water below 37°C (avoiding hot water that may destroy live bacteria) 30 minutes after meals. Start taking capsules 3 days before chemotherapy and continue for 8 weeks. ③ Compliance Management: Nurses conduct weekly phone follow-ups to verify remaining capsules and record any missed doses (good compliance is achieved if ≤2 doses are missed per week). For mild abdominal bloating (score ≤1 point), temporarily reduce dosage to 1 capsule per dose until symptoms resolve, then resume original dosage. (2) Comprehensive Nursing Measures: ① Personalized Dietary Plan: Clinical dietitians (Registered Dietitian) will complete nutritional assessment within 3 days of enrollment using the PG-SGA scale, and formulate dietary plans based on serum albumin levels and baseline gastrointestinal symptoms: A. Severe bloating (Score ≥2): Increase Yam Porridge (200mL daily) and White Radish Soup (150mL daily), while reducing gas-producing foods like legumes and sweet potatoes; B. Diarrhea (Score ≥2): Adopt low-residue diet (e.g., filtered rice soup, steamed egg custard), limit daily dietary fiber to <10g, and avoid milk/lactose-containing products; C. Constipation (Score ≥2): Add Oatmeal Porridge (150g daily) and Celery Stir-fried with Shiitake Mushrooms (200g daily), supplemented with 200mL warm water on an empty stomach each morning. Adjust meals to 5–6 times daily (3 main meals + 2–3 snacks), choosing easily digestible options such as sugar-free yogurt (100mL per snack) and casein-based whey protein powder (15g per snack). ② Abdominal Massage: Perform 30 minutes after dinner. Have the patient lie flat with knees bent and abdomen relaxed. Place your palms around the navel (avoiding the wound area), and massage clockwise with gentle pressure at a rate of 3 revolutions per minute for 15 minutes (totaling 45 rotations). Assist the patient to lie on their left side for 10 minutes after the massage to promote intestinal motility. ③ Traditional Chinese Medicine (TCM) Characteristic Nursing: Acupoint patch therapy is administered weekly on Mondays, Wednesdays, and Fridays using custom-made herbal patches (prepared by grinding 10g Astragalus, 10g Atractylodes, 10g Poria, and 6g Citri Pericarpium in powder, mixing with ginger juice to form a paste, then forming 2cm×2cm medicinal cakes). Apply the patches to the Zusanli (both sides), Zhongwan (CV12), and Tianshu (CV13) acupoints, secured with breathable adhesive tape. Each application lasts 6 hours (9:00 AM to 3:00 PM), and participants are advised to avoid strenuous activities during this period. Remove the patches early if skin redness or itching occurs. ④ Stepwise Exercise Plan: Based on KPS scoring: A. KPS 60-70: Bedside ankle pump exercises (3 sets/day, 10 repetitions per set, 5 seconds per repetition) in weeks 1-2; Sit-up leg lifts (3 sets/day, 8 repetitions per set, lifting 10cm off the bed) in weeks 3-4; Transition to indoor slow walking (initial 5 minutes/day, increasing by 5 minutes weekly, maximum 20 minutes/day) in weeks 5-8. B. KPS 80-100: Indoor slow walking (10 minutes/day) in weeks 1-2; 15 minutes/day in weeks 3-4; 20–30 minutes/day (in two sessions) in weeks 5-8. Exercise intensity should not exceed 170 minus age beats per minute. Stop immediately if abdominal pain or fatigue occurs. ⑤ Symptom Early Warning Management: Distribute the “Gastrointestinal Symptoms Diary” for patients to record daily: abdominal bloating severity (0–3 points), bowel frequency and stool characteristics (Bristol Stool Classification); dietary intake (percentage of recommended amounts) and exercise duration. Nurses review the diary via WeChat or in the clinic on a daily basis. Immediate intervention is required if any of the following occurs:>3 bowel movements per day: increase oral rehydration salts (III) to 2 bags daily, divided into 4 doses; no bowel movement for 2 consecutive days: add wheat fiber granules (3.5g, twice daily); food intake <50% of recommended amount: contact nutritionist to adjust diet, and consider intravenous nutrition (e.g., short peptide preparations, 500mL/day) if necessary.

Both groups received continuous intervention for 8 weeks, during which the time of occurrence of complications, treatment measures, and outcome were recorded in real time; the questionnaire Star was used to collect patients’ subjective symptoms data every Sunday, and two nurses cross-checked and entered the database to ensure the integrity of data (missing value <5%).

### 2.3 Observation indicators

#### 2.3.1 Gastrointestinal function rating scale

The gastrointestinal function assessment was conducted using the MASCC/ESMO (2023) guideline-based gastrointestinal symptom scale, which evaluates four major dimensions—abdominal distension, diarrhea, constipation, and nausea/vomiting—each rated from 0 to 3 points, with higher scores indicating poorer function. For quality of life evaluation, the standardized EORTC QLQ-C30 scoring system was applied, and raw domain scores were converted to a 0–100 scale according to the official scoring manual. In this system, higher scores for functional and overall health domains denote better quality of life, whereas higher symptom scores indicate more severe distress. These methodological additions ensure transparency, reproducibility, and alignment with internationally accepted clinical assessment standards (Herrstedt et al., 2024). The average score was recorded across four dimensions: (1) Abdominal distension: 0 points (no distension), 1 point (mild distension without eating difficulty), 2 points (moderate distension reducing food intake by one-third), 3 points (severe distension causing >1/3 food reduction or requiring gastrointestinal decompression); (2) Diarrhea: 0 points (no diarrhea), 1 point (1-2 loose stools daily), 2 points (3-4 loose stools daily), 3 points (>5 loose stools daily or dehydration); (3) Constipation: 0 points (daily bowel movement), 1 point (every 2-3 days), 2 points (every 4-5 days), 3 points (>6 days without bowel movement or enema required); (4) Nausea and vomiting: 0 points (no nausea/vomiting), 1 point (mild nausea without vomiting), 2 points (moderate nausea with 1-2 daily vomitings), 3 points (severe nausea with ≥3 daily vomitings). The total score ranges from 0 to 12 points, with higher scores indicating poorer gastrointestinal function.

#### 2.3.2 Nutritional status indicators

(1) Serum Albumin (ALB): Measured using the bromophenol green method, with a standard range of 40–55 g/L; (2) Prealbumin (PA): Assessed through the immunoturbidimetric method, with a standard range of 180–380 mg/Lg/L; (3) Body Mass Index (BMI): Calculated using the formula BMI = weight (kg)/height ² (m ²), measured in the morning on an empty stomach while wearing light clothing.

#### 2.3.3 Quality of life assessment

The European Organization for Research and Treatment of Cancer (EORTC) 30-Item Quality of Life Questionnaire (Cronbach’s α = 0.91) (Park et al., 2018) was used to evaluate patients’ quality of life. This assessment encompasses five functional dimensions (physical function, role function, cognitive function, emotional function, and social functioning), three symptom dimensions (fatigue, pain, and nausea/vomiting), and one overall health dimension. Raw scores from each dimension were converted to 0–100 points using standardized formulas. Higher scores in functional and overall health dimensions indicate better quality of life, while higher scores in symptom dimensions indicate more severe symptoms. Patients completed the questionnaire independently at baseline and at 2, 4, and 8 weeks post-intervention, with all questionnaires collected on-site (100% return rate).

#### 2.3.4 Complications and nursing indicators

(1) Complications: The incidence of complications within 8 weeks of intervention was statistically analyzed, including intestinal obstruction (imaging confirmed bowel dilation with air-fluid level), electrolyte disturbances (serum potassium <3.5mmol/L or>5.5mmol/L, serum sodium <135mmol/L or>145mmol/L), and worsening chemotherapy-related diarrhea (diarrhea score ≥3 and persisting>48 hours). (2) Nursing Satisfaction: A self-developed satisfaction scale (Cronbach’s α=0.87) was used to evaluate nursing attitude, professional skills, symptom relief effectiveness, etc., across 8 items. A 5-point scale was adopted, with scores ranging from 1 (very dissatisfied) to 5 (very satisfied). Total scores ≥40 indicated very satisfied, 32–39 indicated satisfied, and scores <32 indicated dissatisfied. Satisfaction rate = (number of very satisfied + number of satisfied)/total cases × 100%.

### 2.4 Statistical methods

Statistical analysis was conducted using SPSS 25.0 software. A one-way ANOVA followed by Tukey’s *post-hoc* test was used for multiple group comparisons. At the same time, the Generalized Estimating Equation (GEE) model was applied to evaluate the time-intervention effects. For normally distributed quantitative data, mean values ± standard deviation were presented, with t-tests employed for inter-group comparisons. Categorical data were expressed as frequencies and percentages (%), with χ² tests used for group comparisons. Ordinal data utilized rank sum tests. An exchangeable correlation matrix was applied, and P <0.05 was considered statistically significant.

## 3 Results

### 3.1 Comparison of general data between the two groups

As shown in **Table 1**, there was no statistically significant difference between the two groups in terms of age, gender, tumor type, chemotherapy regimen, disease course, and other baseline data (P > 0.05), indicating comparable characteristics.

**Table 1:** Baseline clinical characteristics of advanced digestive malignancy patients in the observation (n = 150) and control (n = 150) groups.

Indicator	Observation group (n=150)	Control group (n=150)	*^t/x2^*	*P*
Age (years)	58.73±10.62	57.91±11.38	0.645	0.519
Gender				
Male	89(59.33)	85(56.67)	0.219	0.640
Female	61(40.67)	65(43.33)		
Tumor type				
Gastric cancer	52(34.67)	48(32.00)	0.356	0.837
Colorectal cancer	67(44.67)	72(48.00)		
Esophageal cancer	31(20.66)	30(20.00)		
Chemotherapy regimen				
SOX	52(34.67)	48(32.00)	0.356	0.837
FOLFOX	67(44.67)	72(48.00)		
TP	31(20.66)	30(20.00)		
Disease duration (weeks)	24.38±5.72	23.14±6.69	1.725	0.085
Karnofsky score (points)	75.62±8.37	74.89±9.12	0.722	0.471
BMI(kg/m²)	21.87±3.15	22.13±3.42	0.685	0.494

Data are expressed as mean ± SD or n (%), as appropriate.

### 3.2 Comparison of gastrointestinal function scores between the two groups

As shown in **Table 2**, there was no significant difference (P > 0.05) in the scores and total scores of gastrointestinal function dimensions between the two groups before the intervention. After 2 weeks, 4 weeks, and 8 weeks of intervention, the scores and total scores of each dimension in both groups decreased compared to those before intervention, and the observed group was significantly lower than that of the control group (P < 0.05).

**Table 2:** Gastrointestinal function scores at different intervention time points (before, 2, 4, and 8 weeks) between the two groups.

Indicator	Group	n	Before intervention	2 weeks after intervention	4 weeks after intervention	8 weeks after intervention
Abdominal distension	Observation group	150	1.87±0.63	1.23±0.48	0.86±0.35	0.42±0.21
	Control group	150	1.92±0.58	1.67±0.52	1.35±0.47	0.98±0.36
	*T*		0.715	7.615	10.241	16.456
	*P*		0.475	0.000	0.000	0.000
Diarrhea	Observation group	150	1.73±0.56	1.15±0.42	0.72±0.31	0.35±0.18
	Control group	150	1.68±0.61	1.52±0.47	1.21±0.43	0.87±0.32
	*T*		0.740	7.189	11.321	17.346
	*P*		0.460	0.000	0.000	0.000
Constipation	Observation group	150	1.62±0.51	1.08±0.37	0.65±0.28	0.29±0.15
	Control group	150	1.57±0.55	1.43±0.42	1.12±0.39	0.76±0.29
	*T*		0.816	7.658	11.990	17.631
	*P*		0.415	0.000	0.000	0.000
Nausea and vomiting	Observation group	150	2.15±0.67	1.42±0.53	0.93±0.39	0.51±0.24
	Control group	150	2.21±0.72	1.89±0.61	1.56±0.52	1.13±0.41
	*T*		0.747	7.123	11.871	15.983
	*P*		0.456	0.000	0.000	0.000
Total score	Observation group	150	7.37±1.85	4.88±1.36	3.16±0.97	1.57±0.58
	Control group	150	7.38±1.92	6.51±1.63	5.24±1.45	3.74±1.05
	*T*		0.046	9.404	14.603	22.156
	*P*		0.963	0.000	0.000	0.000

Data are mean ± SD (n = 150 per group).

**Figure 1:**
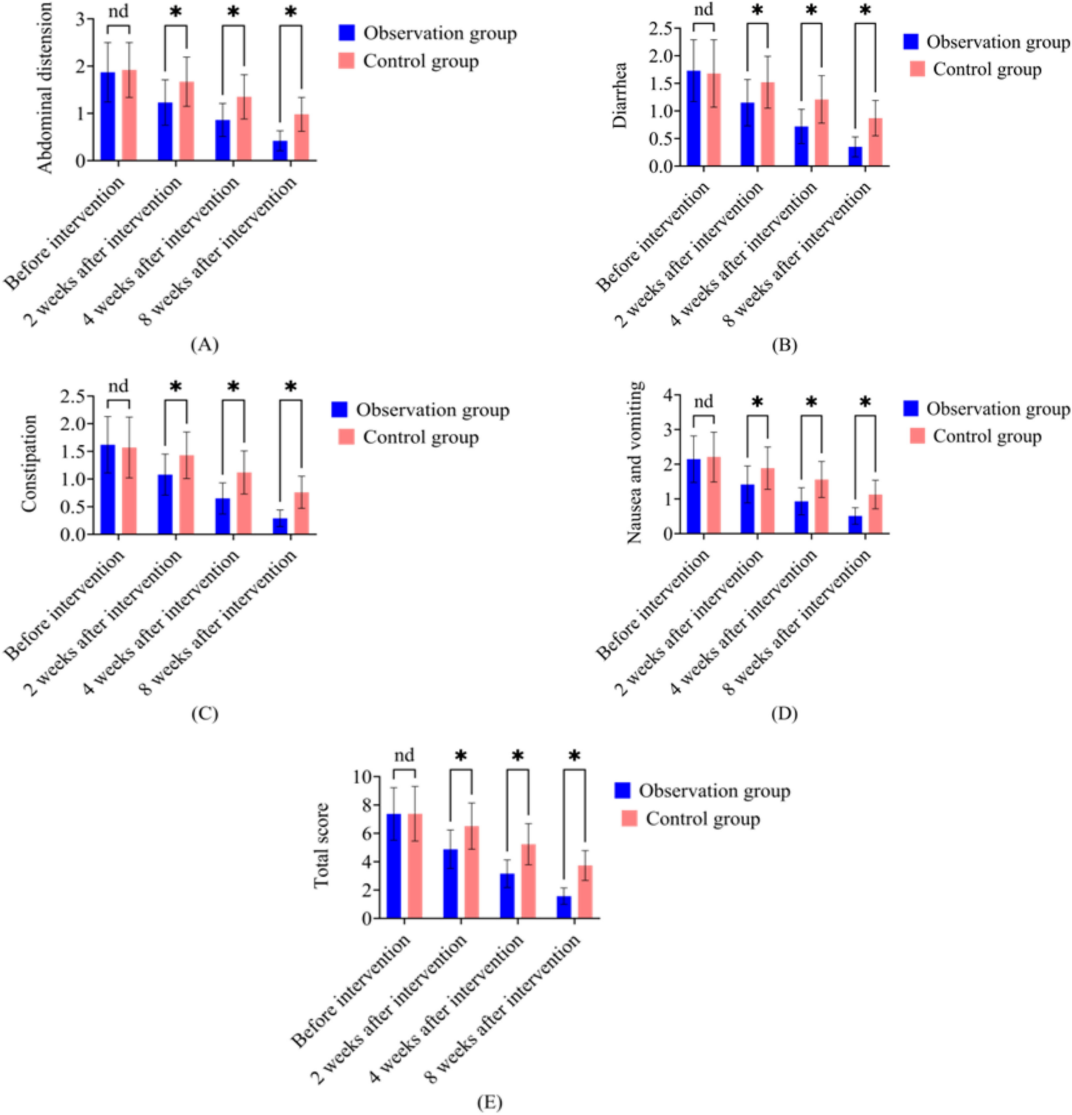
Comparison of gastrointestinal function scores between the observation group and control group at different intervention time points. **(A)** Abdominal distension scores; **(B)** Diarrhea scores; **(C)** Constipation scores; **(D)** Nausea and vomiting scores; **(E)** Total gastrointestinal function scores. Data are presented as mean ± SD. *P < 0.05 indicates statistically significant difference between groups; nd = no significant difference (P > 0.05).

Note: **Figure 1**: Statistical analyses were performed using one-way ANOVA followed by Tukey’s *post-hoc* test for multiple group comparisons. Comparison of gastrointestinal function scores between control and observation groups at different intervention durations (2, 4, and 8 weeks). In all figures, * represents *P* < 0.05, and “nd” denotes no significant difference between groups.

### 3.3 Comparison of nutritional status indexes between the two groups

As shown in **Table 3**, there was no statistically significant difference in serum albumin, prealbumin, and BMI between the two groups before intervention (P > 0.05). After 2, 4, and 8 weeks of intervention, all indicators in the observation group showed significantly higher levels compared to pre-intervention values, and were notably higher than the corresponding levels in the control group (P < 0.05). In contrast, only the BMI in the control group demonstrated a statistically significant difference compared to pre-intervention levels at week 8 (P < 0.05).

**Table 3:** Nutritional indicators (serum albumin, prealbumin, BMI) measured before and after intervention at 2, 4, and 8 weeks in both groups.

Indicator	Group	n	Before intervention	2 weeks after intervention	4 weeks after intervention	8 weeks after intervention
Serum albumin (g/L)	Observation group	150	34.26±3.75	36.82±3.51	39.17±3.28	41.53±2.96
	Control group	150	33.98±3.82	34.56±3.67	35.21±3.54	36.18±3.42
	*t*		0.641	5.451	10.050	14.487
	*P*		0.522	0.000	0.000	0.000
Prealbumin (mg/L)	Observation group	150	186.35±24.72	215.87±22.36	243.62±19.85	276.41±17.53
	Control group	150	184.72±25.36	192.53±23.18	201.37±21.64	218.65±20.37
	*t*		0.564	8.876	17.621	26.323
	*P*		0.573	0.000	0.000	0.000
BMI (kg/m²)	Observation group	150	21.87±3.15	22.36±3.08	22.94±2.97	23.58±2.86
	Control group	150	22.13±3.42	21.28±3.36	22.15±3.29	22.37±3.21
	*t*		0.685	2.902	2.183	3.447
	*P*		0.494	0.004	0.030	0.001

Note: **Figure 2**: Comparison of nutritional indicators (serum albumin, prealbumin, BMI) between the two groups across different intervention periods. Statistical analyses were performed using one-way ANOVA followed by Tukey’s *post-hoc* test for multiple group comparisons. In all figures, * represents *P* < 0.05, and “nd” denotes no significant difference between groups.

**Figure 2:**
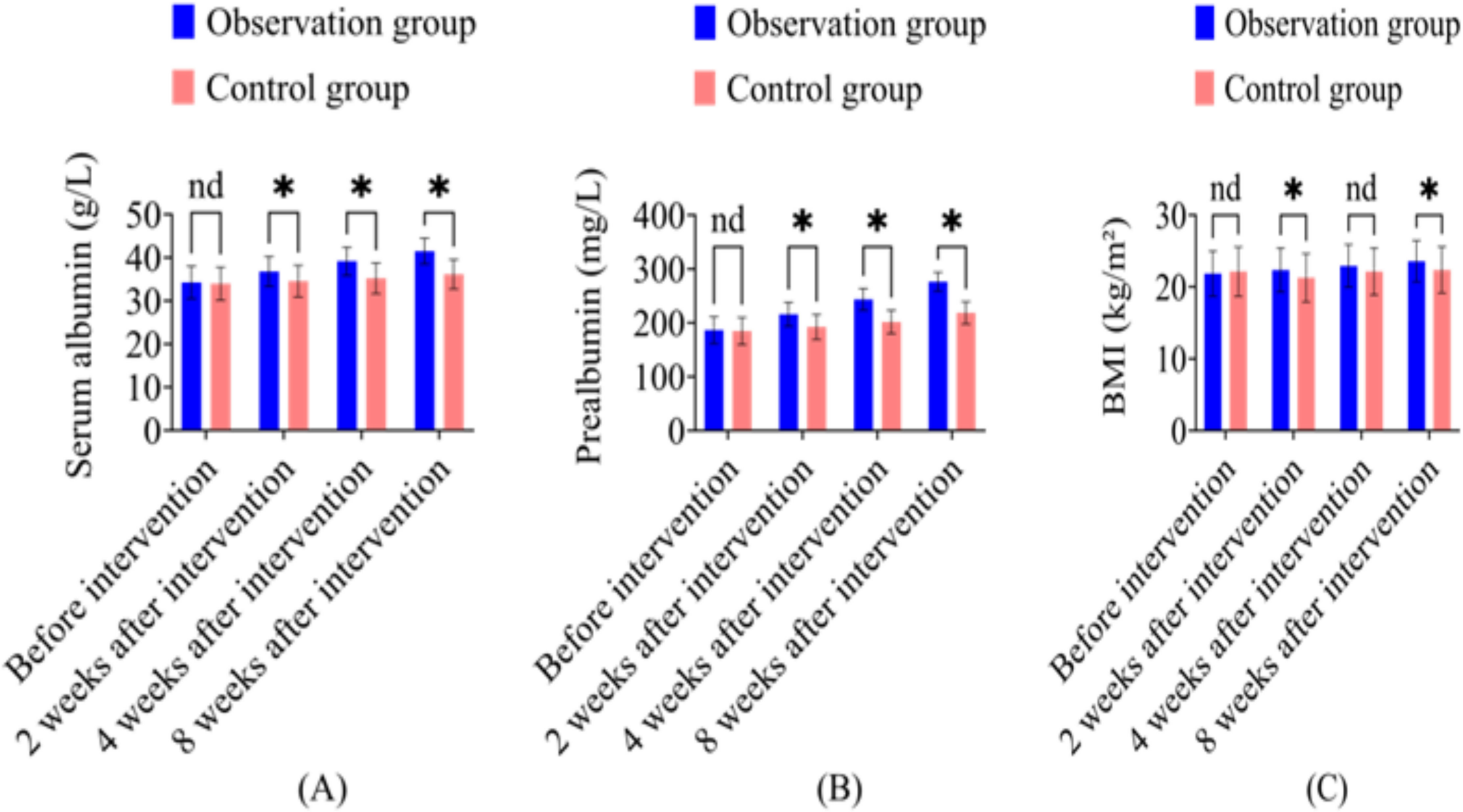
Comparison of nutritional indicators between the observation group and control group at different intervention time points. **(A)** Serum albumin levels (g/L); **(B)** Prealbumin levels (mg/L); **(C)** Body Mass Index (BMI, kg/m²). Data are presented as mean ± SD. *P < 0.05 indicates statistically significant difference between groups; nd = no significant difference (P > 0.05).

### 3.4 Comparison of EORTCQLQ-C30 scores between the two groups

As shown in **Table 4**, there was no statistically significant difference (P>0.05) in the dimensions of EORTCQLQ-C30 between the two groups before intervention. After 2 weeks, 4 weeks, and 8 weeks of intervention, the observation group showed significantly higher scores in the functional dimension and overall health status than the control group, while the symptom dimension score was considerably lower than that of the control group (P < 0.05).

**Table 4:** Comparison of EORTC QLQ-C30 quality-of-life domain scores between the two groups at different time points.

Indicator	Group	n	Before intervention	2 weeks after intervention	4 weeks after intervention	8 weeks after intervention
Physical function	Observation group	150	52.37±8.64	61.52±7.83	69.48±6.75	76.35±5.92
	Control group	150	51.89±8.92	55.26±8.15	58.73±7.64	62.18±7.35
	*t*		0.473	6.784	12.915	18.389
	*P*		0.636	0.000	0.000	0.000
Role function	Observation group	150	48.62±9.15	57.38±8.42	65.27±7.53	72.14±6.86
	Control group	150	47.98±9.36	51.25±8.76	54.63±8.12	58.37±7.64
	*t*		0.599	6.179	11.767	16.425
	*P*		0.550	0.000	0.000	0.000
Cognitive function	Observation group	150	56.38±7.92	63.54±7.26	69.82±6.53	75.64±5.87
	Control group	150	55.76±8.15	58.37±7.54	61.25±7.06	65.42±6.93
	*t*		0.668	6.049	10.914	13.782
	*P*		0.505	0.000	0.000	0.000
Emotional function	Observation group	150	51.24±9.36	59.87±8.64	67.53±7.82	74.26±6.95
	Control group	150	50.78±9.62	53.62±8.95	57.38±8.24	61.54±7.83
	*t*		0.420	6.153	10.943	14.880
	*P*		0.675	0.000	0.000	0.000
Social function	Observation group	150	53.65±8.74	62.18±7.95	69.37±7.26	76.54±6.53
	Control group	150	52.98±8.96	55.73±8.24	59.26±7.83	63.42±7.15
	*t*		0.656	6.899	11.596	16.594
	*P*		0.513	0.000	0.000	0.000
Fatigue	Observation group	150	62.37±9.15	54.26±8.37	46.18±7.54	38.25±6.73
	Control group	150	63.12±9.36	60.53±8.74	56.37±8.12	51.26±7.54
	*t*		0.702	6.346	11.263	15.766
	*P*		0.483	0.000	0.000	0.000
Pain	Observation group	150	58.62±8.54	51.37±7.92	43.25±7.16	36.18±6.34
	Control group	150	59.17±8.76	56.24±8.15	52.37±7.64	47.52±6.98
	*t*		0.551	5.248	10.668	14.729
	*P*		0.582	0.000	0.000	0.000
Nausea and vomiting	Observation group	150	65.38±9.26	56.17±8.54	47.25±7.63	39.18±6.82
	Control group	150	64.92±9.53	62.38±8.96	58.17±8.35	52.36±7.64
	*t*		0.424	6.145	11.824	15.762
	*P*		0.672	0.000	0.000	0.000
Total score	Observation group	150	46.25±8.74	54.38±7.96	62.17±7.25	69.36±6.54
	Control group	150	45.78±8.96	48.62±8.25	52.37±7.83	56.18±7.32
	*t*		0.460	6.154	11.248	16.445
	*P*		0.646	0.000	0.000	0.000

Note: **Figure 3**: Comparison of EORTC QLQ-C30 quality-of-life scores between control and observation groups at various time points. Statistical analyses were performed using one-way ANOVA followed by Tukey’s *post-hoc* test for multiple group comparisons. In all figures, * represents *P* < 0.05, and “nd” denotes no significant difference between groups.

**Figure 3:**
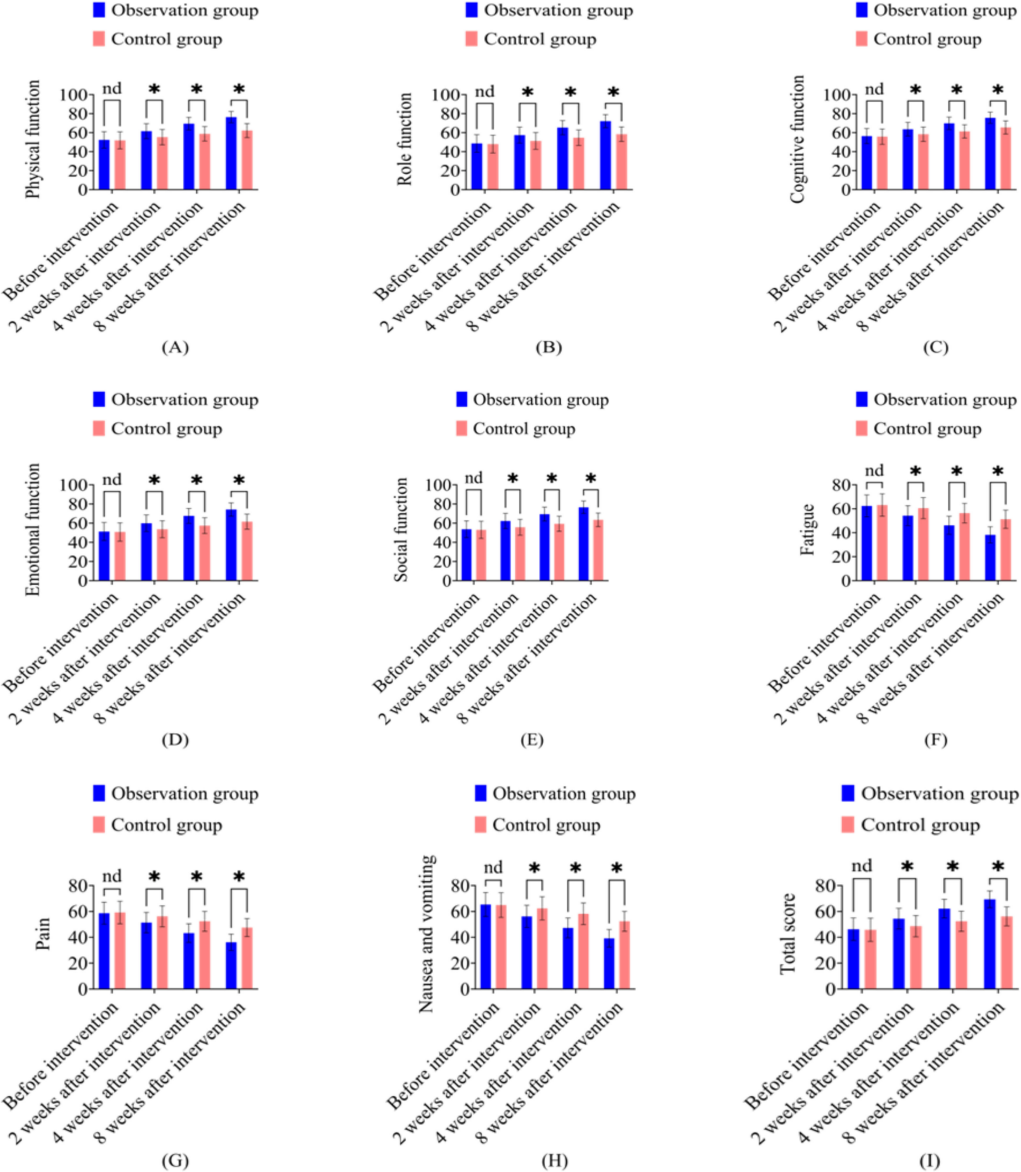
Comparison of EORTC QLQ-C30 quality-of-life scores between the observation group and control group at different intervention time points. **(A)** Physical function; **(B)** Role function; **(C)** Cognitive function; **(D)** Emotional function; **(E)** Social function; **(F)** Fatigue; **(G)** Pain; **(H)** Nausea and vomiting; **(I)** Total score. Data are presented as mean ± SD. *P < 0.05 indicates statistically significant difference between groups; nd = no significant difference (P > 0.05).

### 3.5 The GEE model analyzes the influence of intervention methods on relevant indicators

Using intervention mode (observation group/control group) as the independent variable, time (after intervention, 2 weeks, 4 weeks, 8 weeks) and their interaction as independent variables, and each observation index as the dependent variable, a GEE model was constructed. The data were fitted using an exchange-correlation matrix. The results showed that:

For the total gastrointestinal function score, the main effect β value of the observation group was negative (β=-1.872), and the interaction β value between time and observation group was also negative (β=-0.754), indicating that with the prolongation of intervention time, the improvement of gastrointestinal function in the observation group was significantly greater than that in the control group (P<0.001).

For the nutritional status indicators (serum albumin, prealbumin and BMI), the main effect β values of the observation group were all positive (β=2.157,8.632,0.524), and the interaction β values of time and observation group were also positive (β=0.862,6.357,0.318), indicating that the increase of nutritional indicators in the observation group was significantly better than that in the control group over time (P<0.01).

For the total score of EORTCQLQ-C30, the main effect β value of the observation group was positive (β=12.563), and the interaction β value of time and observation group was also positive (β=8.742), indicating that the improvement of quality of life in the observation group was significantly greater than that in the control group (P<0.001) (**Table 5**).

**Table 5:** Results of generalized estimating equation (GEE) model evaluating time-intervention effects on gastrointestinal, nutritional, and quality-of-life indices.

Indicator	Variable	*β*	*SE*	Waldχ²	*P*
Total score of gastrointestinal function	(Intercept)	7.368	0.425	299.637	<0.001
	Observation group	-1.872	0.563	11.085	<0.001
	Control group	0*	-	-	-
	Time	-0.963	0.172	31.572	<0.001
	Time × Observation group	-0.754	0.216	12.183	<0.001
	Time × Control group	0*	-	-	-
Serum albumin	(Intercept)	34.125	0.736	215.386	<0.001
	Observation group	2.157	0.683	9.872	0.002
	Control group	0*	-	-	-
	Time	1.036	0.215	23.085	<0.001
	Time × Observation group	0.862	0.264	10.637	0.001
	Time × Control group	0*	-	-	-
Prealbumin	(Intercept)	185.624	5.372	118.635	<0.001
	Observation group	8.632	2.754	9.863	0.002
	Control group	0*	-	-	-
	Time	5.247	1.368	14.725	<0.001
	Time × Observation group	6.357	1.982	10.157	0.001
	Time × Control group	0*	-	-	-
BMI	(Intercept)	21.963	0.685	101.572	<0.001
	Observation group	0.524	0.213	6.025	0.014
	Control group	0*	-	-	-
	Time	0.268	0.097	7.632	0.006
	Time × Observation group	0.318	0.125	6.457	0.011
	Time × Control group	0*	-	-	-
EORTC QLQ-C30 total score	(Intercept)	385.624	12.753	93.625	<0.001
	Observation group	12.563	3.187	15.632	<0.001
	Control group	0*	-	-	-
	Time	15.327	2.864	28.753	<0.001
	Time × Observation group	8.742	2.156	16.527	<0.001
	Time × Control group	0*	-	-	-

0* indicates that the parameter is a reference value and is set to 0.

### 3.6 Comparison of complications and nursing indicators between the two groups

The incidence of non-infectious chemotherapy-related complications—including intestinal obstruction, electrolyte disturbance, and aggravated chemotherapy-induced diarrhea—was markedly lower in the observation group (8.7%) compared to the control group (19.3%) (*P* < 0.05). These findings indicate that the combined probiotic and comprehensive nursing intervention effectively mitigated treatment-related gastrointestinal toxicity and improved overall clinical stability (**Table 6**).

**Table 6:** Comparison of complication rates and nursing satisfaction levels between the observation and control groups.

Indicator	Category	Observation group (n=150)	Control group (n=150)	*x2/Z*	*P*
Complications	Intestinal obstruction	5(3.33)	16(10.67)		0.04
	Electrolyte disturbance	8(5.33)	23(15.33)		
	Aggravated chemotherapy-induced diarrhea	2(1.33)	13(8.67)		
	Total incidence	15(10.00)	52(34.67)		
Nursing satisfaction	Very satisfied	98(65.33)	67(44.67)	3.473	0.001
	Satisfied	30(20.00)	47(31.33)		
	Dissatisfied	22(14.67)	36(24.00)		
	Satisfaction rate	128(85.33)	114(76.00)	4.189	0.041

Note: **Figure 4**: Radar chart showing incidence and distribution of complications in both groups. Statistical analyses were performed using one-way ANOVA followed by Tukey’s *post-hoc* test for multiple group comparisons. In all figures, * represents *P* < 0.05, and “ns” denotes no significant difference between groups.

**Figure 4:**
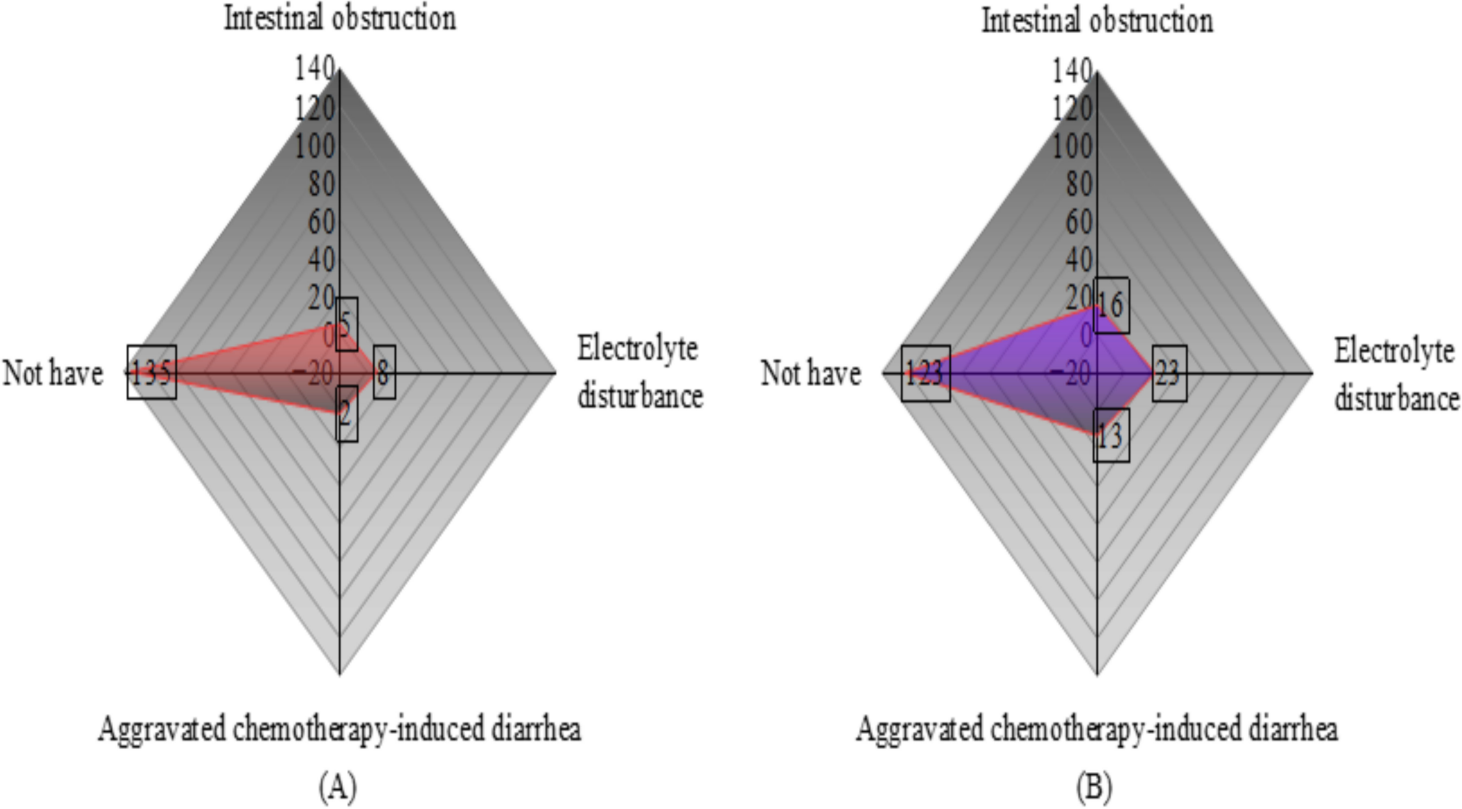
Radar chart showing the incidence of complications in both groups. **(A)** Observation group; **(B)** Control group. The three axes represent intestinal obstruction, electrolyte disturbance, and aggravated chemotherapy-induced diarrhea. Numbers indicate the count of patients in each category.

Note: **Figure 5**: Radar chart representing overall nursing satisfaction rates in both groups. Statistical analyses were performed using one-way ANOVA followed by Tukey’s *post-hoc* test for multiple group comparisons.

**Figure 5:**
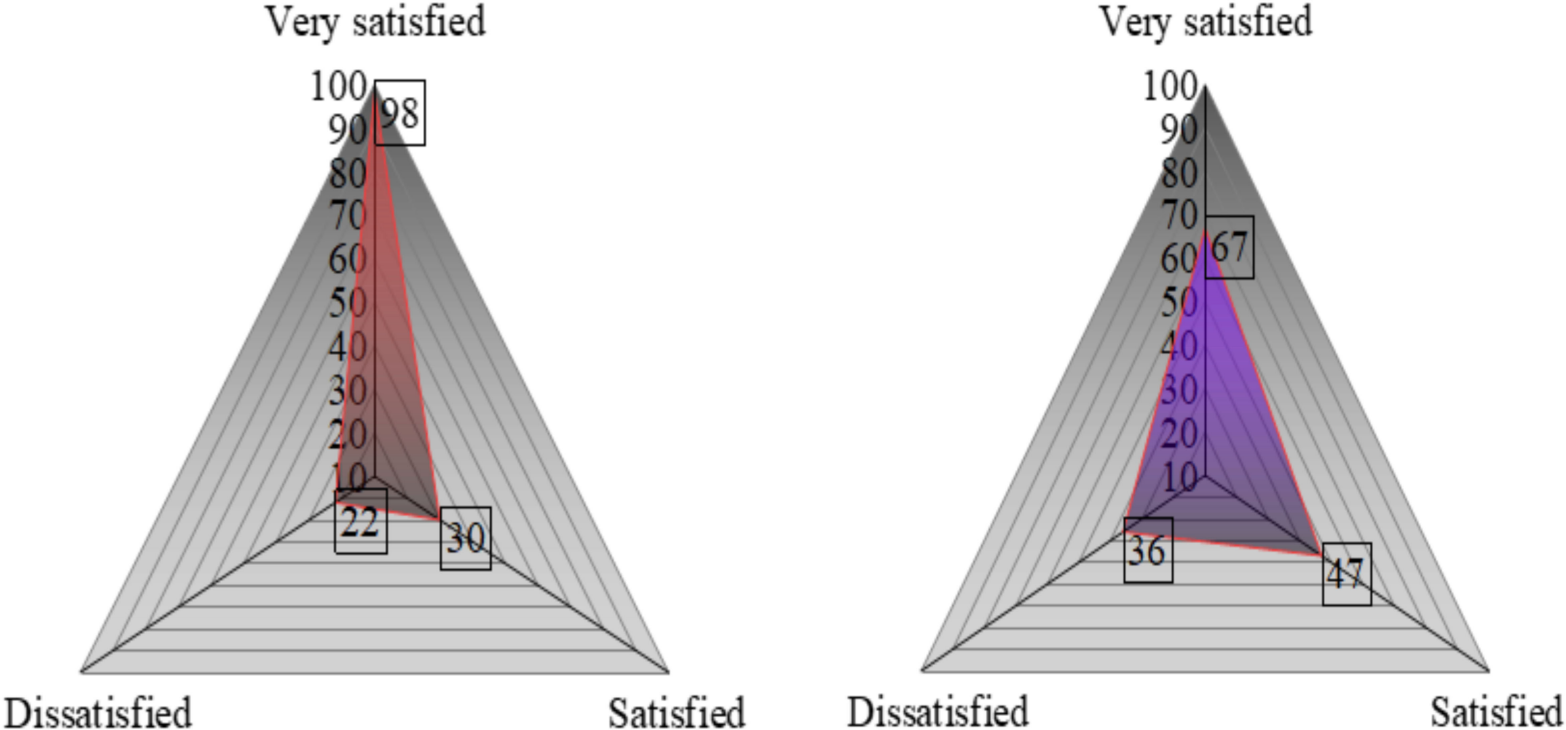
Two sets of satisfaction radar charts.

## 4 Discussion

The present study provides robust clinical evidence that probiotic-assisted comprehensive nursing interventions can significantly improve the gastrointestinal function, nutritional status, and quality of life of patients with advanced digestive malignancy undergoing chemotherapy. The results are supported by well-established biological mechanisms involving gut microbiota modulation, intestinal barrier repair, and immune regulation. Probiotics, such as *Bifidobacterium* and *Lactobacillus acidophilus*, restore the microbial balance disrupted by chemotherapeutic agents, enhance epithelial tight-junction protein expression (occludin, claudin-1), and reduce mucosal inflammation by suppressing pro-inflammatory cytokines, including TNF-α and IL-6.

Gastrointestinal dysfunction in Advanced digestive malignancy patients during chemotherapy results from multiple factors: direct toxicity of chemotherapeutic agents to intestinal mucosa, compromised gut barrier function due to microbial imbalance, and motility disorders caused by neuroendocrine dysfunction (Panc Hosein et al., 2020; Lee et al., 2014; Nakayama et al., 2022). Traditional nursing approaches, which focused on symptomatic management, failed to address the root cause of gut microbiota imbalance, resulting in persistently high recurrence rates of symptoms (Zhuang et al., 2021). In this study, the intervention group received Bifidobacterium Triple Live Capsules (containing Bifidobacterium, Lactobacillus acidophilus, and Enterococcus faecalis). Results showed that after two weeks of treatment, all gastrointestinal dimensions (abdominal distension, diarrhea, constipation, nausea, and vomiting) and overall scores significantly decreased compared to the control group, with advantages persistently expanding over time (P<0.001). This outcome correlates with probiotics’ multi-mechanism effects: Probiotics competitively inhibit pathogenic bacteria (E. coli, Clostridium difficile) adhesion and proliferation, reducing endotoxin release and thereby decreasing intestinal inflammation (Pazhoohan et al., 2020); Dominant bacterial groups like Bifidobacterium enhance tight junction proteins (occludin, claudin) in intestinal epithelial cells, strengthening mucosal integrity and reducing infection risks (Trejo et al., 2006); Probiotic metabolites (e.g., short-chain fatty acids) stimulate neurotransmitter release (e.g., serotonin), improving peristalsis and alleviating constipation and bloating (Li et al., 2024).

Notably, the observation group received comprehensive nursing interventions (including personalized dietary plans, abdominal massage, and TCM acupressure patches) alongside probiotic supplementation. This combined approach likely amplified the effects on gastrointestinal improvement through synergistic interactions. Abdominal massage stimulates colonic peristalsis via clockwise mechanical stimulation, creating synergistic effects with probiotics ‘motility regulation. TCM Apply enhances spleen-stomach digestive functions through meridian pathways, forming a dual regulatory mechanism of “microecology-TCM function” with probiotics (Li et al., 2024). GEE model analysis revealed a time-intervention interaction β value of -0.754 for the observation group’s gastrointestinal function score (P < 0.001), demonstrating that the comprehensive intervention’s dynamic improvement effect significantly outperformed conventional care over time. This confirms the necessity of multidimensional interventions.

Malnutrition is a common complication in Patients with Advanced Gastrointestinal Malignancy, with an incidence rate of 25%-40% (Narimatsu and Yaguchi, 2022). Gastrointestinal dysfunction serves as the core trigger for malnutrition— Abdominal distension, nausea, and vomiting reduce food intake, while diarrhea and malabsorption directly impair nutrient utilization, ultimately creating a vicious cycle of “insufficient intake-absorption impairment-increased consumption” (Jabłońska, 2025). In this study, the intervention group showed significantly higher nutritional indicators (serum albumin, prealbumin, and BMI) after 8 weeks of treatment compared to the control group. Specifically, serum albumin increased from baseline 34.26g/L to 41.53g/L, and prealbumin rose from 186.35mg/L to 276.41mg/L (P values <0.001). In contrast, the control group showed only a slight increase in BMI after 8 weeks of intervention (P = 0.001). The key difference lies in how improved gastrointestinal function enhances nutrient absorption. In the observation group, alleviating gastrointestinal symptoms directly boosted patients’ appetite, while customized dietary plans (e.g., adding Chinese yam porridge for bloating patients and increasing dietary fiber for constipation sufferers) further optimized targeted nutrition delivery (Jo et al., 2017). Probiotics also play dual roles: Lactobacillus acidophilus secretes lactic acid enzymes that improve lactose tolerance in individuals with lactose intolerance. At the same time, Bifidobacterium enhances the absorption of nutrients such as the vitamin B complex and calcium (Milani et al., 2017). Chronic low-grade inflammation caused by an imbalance in gut microbiota accelerates protein catabolism. Probiotics suppress pro-inflammatory factors (e.g., TNF-α and IL-6) to reduce muscle protein breakdown, thereby maintaining stable body mass index (Radford-Smith and Anthony, 2023).

Improving nutritional status not only affects patients’ quality of life but also significantly impacts their tolerance to chemotherapy. Studies show that a 1 g/L increase in serum albumin levels reduces the risk of chemotherapy discontinuation by 12% (Chang et al., 2023). In this study, the incidence of non-infectious complications (e.g., intestinal obstruction and electrolyte imbalances) in the observation group was notably lower than in the control group, which is directly linked to optimized nutritional metrics. A well-nourished state enhances intestinal mucosal repair capacity while reducing the risks of bowel dilation and electrolyte imbalance.

Furthermore, the quality of life for advanced cancer patients is influenced by multiple factors, including physical symptoms, psychological state, and social functioning. Using the EORTC QOLC-30 scale in this study, we observed that after 8 weeks of intervention, the observation group demonstrated significantly higher scores across dimensions, including physical function, role performance, and emotional well-being, compared to the control group. The overall health score increased from 46.25 to 69.36 points (P<0.001). Key contributing factors included: reduced gastrointestinal symptoms (e.g., nausea, vomiting, diarrhea) which markedly decreased physical distress, while improved accompanying symptoms like pain and fatigue further enhanced daily activity capacity; elevated serum albumin levels not only reflected nutritional status but also correlated with patients ‘physical reserve, with stable KPS scores in the observation group providing a foundation for social engagement and role maintenance; a stepwise exercise program in comprehensive care enhanced patients’ self-efficacy, while cultural recognition of TCM-specific nursing practices (such as acupoint patch application) may alleviate resistance to Western medical treatment, indirectly improving emotional functioning (Wang et al., 2025).

The GEE model showed that the interaction between time and intervention of the total quality of life score in the observation group was β8.742 (P<0.001), indicating that with the extension of intervention time, the improvement effect of the comprehensive plan on quality of life gradually amplified, which was consistent with the cumulative effect of symptom relief and nutrition improvement.

However, the limitations of this study are as follows: ① The sample was collected from a single center, which may lead to selection bias; ② The specific changes in gut microbiota diversity (e.g., Bifidobacterium/Escherichia coli ratio) were not monitored, making it difficult to quantify the impact of probiotics on microbial structure; ③ The follow-up period was only 8 weeks, and long-term effects (such as complication incidence after 6 months) require further validation.

Despite these limitations, the clinical translational value of this study remains noteworthy. The probiotic formulation (Bifidobacterium Triple Live Capsules), a clinically established medication, is combined with easily implementable nursing measures (such as abdominal massage and acupoint patching) that are cost-effective and readily adoptable in primary care facilities. The nutritional assessment and symptom grading management based on the PG-SGA scale provide a replicable protocol for precision care in advanced cancer patients. Future research could explore synergistic applications of probiotics and prebiotics (e.g., fructooligosaccharides) to enhance emotional well-being and treatment adherence through the “microbiota-gut-brain axis” pathway (Nakayama et al., 2022).

In conclusion, the comprehensive nursing protocol incorporating probiotic interventions has demonstrated clinical efficacy in disrupting the pathological cycle of Advanced digestive Malignancy Patients during chemotherapy through three key mechanisms: regulating gut microbiota, improving gastrointestinal function, and optimizing nutritional status. This study confirms its significant clinical value. Future research should focus on multi-center, long-term follow-up studies to further validate its applicability across different tumor types and chemotherapy regimens, thereby providing more precise evidence-based support for oncology nursing care.

## 5 Conclusion

This study demonstrates that integrating probiotic intervention with comprehensive nursing care significantly enhances the clinical outcomes of patients with advanced digestive malignancies undergoing chemotherapy. The combined approach effectively improved gastrointestinal function, alleviating symptoms such as bloating, diarrhea, constipation, and nausea/vomiting. It also optimized nutritional status, as reflected by elevated serum albumin, prealbumin, and BMI levels, while reducing the incidence of non-infectious complications and improving overall quality of life and nursing satisfaction. These findings highlight the synergistic benefits of probiotics and personalized nursing interventions in breaking the cycle of chemotherapy-induced gastrointestinal dysfunction and malnutrition. The proposed nursing protocol is practical, evidence-based, and adaptable for clinical application in oncology settings. Future research should focus on validating these results through multicenter trials, exploring the microbiome-level mechanisms underlying the observed effects, and assessing the long-term impact of probiotic-based nursing strategies on patient survival and treatment adherence.

## Data Availability

The original contributions presented in the study are included in the article/supplementary material. Further inquiries can be directed to the corresponding author.
